# NiTi-Layered Double Hydroxide@Carbon Nanotube as a Cathode Material for Chloride-Ion Batteries

**DOI:** 10.3390/nano13202779

**Published:** 2023-10-17

**Authors:** Lu Zou, Shijiao Sun, Chang Zhang, Xiangyu Zhao

**Affiliations:** 1 State Key Laboratory of Materials-Oriented Chemical Engineering, College of Materials Science and Engineering, Nanjing Tech University, Nanjing 211816, China; 202061203195@njtech.edu.cn (L.Z.); 202162103016@njtech.edu.cn (C.Z.); xiangyu.zhao@njtech.edu.cn (X.Z.); 2 Jiangsu Collaborative Innovation Center for Advanced Inorganic Functional Composites, Nanjing Tech University, 30 Puzhu South Road, Nanjing 211816, China

**Keywords:** layered double hydroxide, carbon nanotubes, chloride ion batteries

## Abstract

Chloride-ion batteries (CIBs) are one of the promising candidates for energy storage due to their low cost, high theoretical energy density and high safety. However, the limited types of cathode materials in CIBs have hindered their development. In this work, a NiTi-LDH@CNT composite is prepared using a reverse microemulsion method and applied in CIBs for the first time. The specific surface area and the pore volume of the obtained NiTi-LDH@CNT composites can reach 266 m^2^ g^−1^ and 0.42 cm^3^ g^−1^, respectively. Electrochemical tests indicate that the composite electrode delivers a reversible specific capacity of 69 mAh g^−1^ after 150 cycles at a current density of 100 mA g^−1^ in 0.5 M PP_14_Cl/PC electrolyte. Ni^2+^/Ni^3+^ and Ti^3+^/Ti^4+^ valence changes during electrochemical cycling are demonstrated by X-ray photoelectron spectroscopy (XPS), while reversible migration of Cl^−^ is revealed by ex-situ EDS and ex-situ XRD. The stable layered structure and abundant valence changes of the NiTi-LDH@CNT composite make it an exceptional candidate as a cathode material for CIBs.

## 1. Introduction


(1) $$\mathrm{Anode}:\;M^{\prime }+xCl^{-}⇌\;M^{\prime }Cl_{x}+xe^{-}$$

(2) $$\mathrm{Cathode}:\;MCl_{x}+xe^{-}⇌M+xCl^{-}$$

Due to the increasing demand for various energy resources, how to efficiently store and convert energy is the key to achieving sustainable development [1,2]. However, the large-scale development of lithium-ion batteries, which are still the mainstream batteries for commercialization, is hindered by high cost and the uneven distribution of lithium metal resources [3,4]. To overcome these limitations, chloride-ion batteries (CIBs) have been proposed as a potential alternative to lithium-ion batteries. The working principle of the rechargeable CIBs can be described as follows:

The basic working principle of the CIBs involves the use of the chloride ion as a shuttle between cathode and anode in a chloride-containing electrolyte. During discharge, chloride ions migrate from the cathode and are transported to the anode through the electrolyte. Upon charging, the reverse process occurs. CIBs are characterized by low cost due to abundant chloride resources and a high theoretical volumetric energy density of 2500 Wh L^−1^ [5,6]. Moreover, CIBs offer significant advantages in terms of safety due to their unique dendritic-free nature. These features make them highly suitable for large-scale energy storage applications [7]. However, the practical application of CIBs has been hindered by the lack of suitable high-performance cathode materials. Thus, the development of cathode materials that can efficiently store and release Cl^−^ is essential for the development of high-performance CIBs [8,9,10,11].

Many materials including metal chlorides, metal oxychlorides, chloride ion-doped conductive polymers, layered double hydroxides (LDHs) and organic electrode material have been developed as cathode materials for CIBs. In the first study, commercial CoCl_2_, VCl_3_, and BiCl_3_ [5] materials were used to demonstrate proof of principle for CIBs. The capacity loss of the batteries is very serious due to the easy dissolution of metal chlorides in the electrolyte. Later, metal oxychlorides such as BiOCl [12], FeOCl [13,14], VOCl [6], and Sb_4_O_5_Cl_2_ [15] with stable structure were explored as cathode materials for CIBs. Compared with metal chloride, metal oxychlorides exhibited excellent cycle stability and long cycle life. Subsequently, conductive polymer cathode materials (PPyCl_0.33_ [16], PANICl_0.25_ [17]) attracted significant attention. Due to the low volume change during discharging and charging, the capacity retention of the conductive polymer cathodes was excellent. However, the theoretical capacity of conductive polymer cathodes was generally low.

Layered double hydroxide (LDH) is an inorganic two-dimensional material that contains anions and has a structural formula of [$M_{1-x}^{\mathrm{II}}M_{x}^{\mathrm{III}}$ (OH)_2_]^z+^(A^n−^)_z/n_·yH_2_O (where M^II^ and M^III^ represent divalent and trivalent metals, respectively. A^n−^ represents interlaminar anions that compensate for positively charged octahedral metal layers) [18]. The LDH has a 2D diffusion channel that allows for stable insertion/deinsertion of Cl^−^ during electrochemical cycling without significant volume change. Several LDHs with interlayer spacing between 0.761 nm and 0.783 nm (much larger than the ionic radius of Cl^−^ 0.181 nm), including CoFe-Cl LDH [19], NiMn-Cl LDH [20], Ni_2_V_0.9_Al_0.1_-Cl LDH [21], NiFe-Cl LDH [22], CoNi-Cl LDH [23] and NiTi-Cl LDH [24], have been investigated as cathode materials for CIBs (Table 1). In 2019, CoFe-Cl LDH [19] was prepared by a co-precipitation method and reported for the first time as a CIB cathode material. At a current density of 100 mA g^−1^, a reversible specific capacity of 160 mAh g^−1^ was maintained after 100 cycles in 0.5 M Bpy_14_Cl-PP_14_TFSI-PC electrolyte. In 2020, NiMn-Cl LDH [20] was also synthesized by a co-precipitation method and employed in a CIB system. At a current density of 50 mA g^−1^, a reversible specific capacity of 130 mAh g^−1^ was obtained after 150 cycles in 0.5 M Bpy_14_Cl-PC electrolyte. Soon after, Ni_2_V_0.9_Al_0.1_-Cl LDH [21] was synthesized by a hydrothermal method followed by an ion-exchange process. At a current density of 200 mAg^−1^, Ni_2_V_0.9_Al_0.1_-Cl LDH cathode delivered a reversible specific capacity of 113.8 mAh g^−1^ after 1000 cycles in 1 M Bpy_14_Cl-PP_14_TFSI-PC electrolyte. In the same year, NiFe-Cl LDH [22] was also synthesized by a hydrothermal method combined with an anion-exchange process. At a current density of 100 mA g^−1^, the NiFe-Cl LDH cathode exhibited a reversible specific capacity of 130 mAh g^−1^ after 100 cycles in 0.5 M Bpy_14_Cl-PC electrolyte. Additionally in 2020, CoNi-Cl LDH [23] was synthesized by ion-exchange of as-prepared CoNi-Br LDH. A reversible specific capacity of 83 mAh g^−1^ remained after 50 cycles in 0.5 M Bpy_14_Cl-PC/[PP_14_][NTf_2_] electrolyte. In 2023, Han et al. [24] performed high-throughput screening computation of layered double hydroxides as cathodes for chloride ion batteries. They found that Ti-containing LDHs were screened as the most promising cathodes through the theoretical voltage calculation. Although some progress has been made in LDHs, the current LDHs for CIBs usually possess much larger particle size (micro size) and consist of multilayers. Additionally, the preparation process for LDHs is usually complicated, N_2_ gas protection is usually mandatory for the conventional co-precipitation method.

Moreover, to the best of our knowledge, all the reported cathode materials contain elemental chlorine. Herein, a new chlorine-free cathode material, NiTi-LDH@CNT, was prepared by a facile reverse microemulsion method and developed as a cathode material for CIBs. Unlike the conventional co-precipitation method, the preparation of LDH by the reverse microemulsion method does not require the passage of N_2_ gas throughout the experiment. The specific surface area and pore volume of the NiTi-LDH@CNTs prepared by the reverse microemulsion method are as high as 266 m^2^ g^−1^ and 0.42 cm^3^ g^−1^, respectively, which is beneficial for sufficient contact between the electrode and electrolyte and promotes the electrochemical performance. In the following, we will discuss the synthesis, characterization, and electrochemical performance of NiTi-LDH@CNTs as a CIB electrode material.

## 2. Experimental

### 2.1. Synthesis of NiTi-LDH and NiTi-LDH@CNTs

To prepare NiTi-LDH, a reverse microemulsion method was used [25,26,27]. A mixture of 1.1 mL water, 50 mL iso-octane, and 1.80 g SDS was added to a three-mouth flask with constant stirring. This was followed by the addition of 1.5 mL 1-butanol until a transparent reverse microemulsion was formed. Ni(NO_3_)_2_·6H_2_O (0.004 mol) and TiCl_4_ (0.001 mol) were then added to the above microemulsion, followed by adding 1.2 g urea to form a blue-green solution. The mixture was heated in an oil bath at 110 °C and continuously stirred for 27 h. The resulting product was then centrifuged and washed several times using a mixed solvent of ethanol and water (volume ratio of 1:1), and dried in a vacuum oven at 60 °C for 24 h.

To synthesize NiTi-LDH@CNTs, the same reverse microemulsion method was used with the addition of 50 mg carbon nanotubes to the microemulsion. The mixture was sonicated for 1 h before the addition of urea.

### 2.2. Materials Characterization

X-ray diffraction (XRD) data were acquired from a Rigaku SmartLab diffractometer with Cu Kα radiation (λ = 0.15418 nm). The Fourier transform infrared spectroscopy (FTIR) was carried out using a Thermo Scientific Nicolet iS20 Spectrometer in the wave number range from 400 to 4000 cm^−1^. Thermogravimetric analyses (TGA) were carried out at a ramp rate of 10 °C min^−1^ under an air atmosphere using the NETZSCH STA449F3 instrument. The morphologies of the samples were monitored using a field-emission scanning electron microscope (ZEISS Ultra 55) at an accelerating voltage of 5 kV, the compositions of the samples were explored by an energy dispersive X-ray spectrometer attachment (Oxford Instrument). Nitrogen adsorption/desorption measurements were performed on a Micromeritics 3Flex surface characterization analyzer. The carbon content in the NiTi-LDH@CNTs was determined by an Elementar unicube elemental analyzer. X-ray photoelectron spectroscopy (XPS) measurements were carried out on a Thermo Scientific K-Alpha spectrometer with Al Kα X-rays as the excitation source.

### 2.3. Preparation of the Anode

Since the NiTi-LDH herein is a Cl¯ free material, the LiCl/Li anode must be prepared prior to electrochemical characterization. For the preparation of LiCl/Li anode, the Li‖FeOCl battery was first assembled with a mixture of 0.5 M PP_14_Cl in PP_14_TFSI as electrolyte. The FeOCl material was prepared by a thermal decomposition method previously reported [28]. The FeOCl electrode was prepared by mixing 80 wt% FeOCl, 10 wt% acetylene black and 10 wt% polyvinylidene fluoride (PVDF) in N-methyl-2-pyrrolidone (NMP). The obtained slurry was cast onto a graphite foil current collector with a thickness of 150 μm. After drying under vacuum at 80 °C for 12 h, a round electrode with a diameter of 19 mm was punched out. The load weight of FeOCl is ~3 mg. Then the assembled Li‖FeOCl battery was subjected to full discharge to 1.6 V at 10 mA g^−1^. After the discharge is completed, the battery was disassembled and the lithium foil was wiped with clean glass fiber to remove the residual electrolyte. 

### 2.4. Electrochemical Measurements

CR2032 coin cells were assembled with the as-prepared NiTi-LDH or NiTi-LDH@CNT as the cathode material, Celgard 2400 membrane as the separator, and the as-prepared LiCl/Li as the anode. The cathode was prepared by mixing 60 wt% NiTi-LDH or NiTi-LDH@CNTs, 30 wt% Super P, and 10 wt% PVDF in NMP. The obtained slurry was spread onto a stainless-steel foil current collector with a thickness of 150 μm. After drying under vacuum, a round electrode with a diameter of 12 mm was punched out. The electrolyte was a mixture of 0.5 M PP_14_Cl in PC. The specific capacity was calculated based on the mass of NiTi-LDH. 

Electrochemical impedance spectroscopy (EIS, 100 kHz to 10 mHz, 10 mV) and cyclic voltammetry (CV, 1.2 to 3 V, 0.1 mV s^−1^) measurements were performed on the BioLogic (VMP3) electrochemical workstation. The EIS data was fitted with Zview software. Galvanostatic discharge and charge measurements were conducted on the Neware battery test system in a voltage range of 1.2–3 V.

## 3. Results

### 3.1. Structural Characterization and Compositional Analysis of NiTi-LDH and NiTi-LDH@CNT Materials

**Figure 1:**
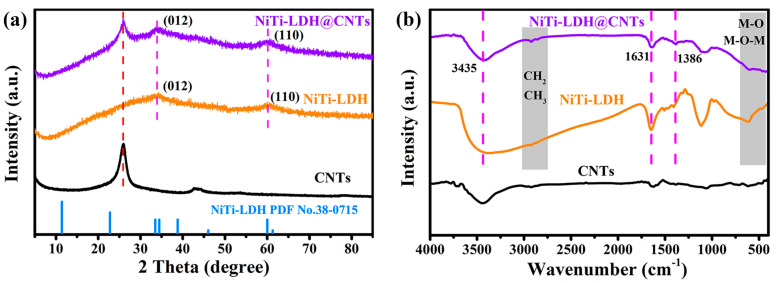
(**a**) XRD patterns and (**b**) FT-IR spectra of the CNT, NiTi-LDH and NiTi-LDH@CNT materials.

The XRD patterns of NiTi-LDH, NiTi-LDH@CNTs and CNTs are shown in Figure 1a. The diffraction peaks of NiTi-LDH and NiTi-LDH@CNT samples prepared through the reverse microemulsion method are notably broad and weak, indicating the poor crystallinity of the synthesized material. The NiTi-LDH sample herein prepared following the procedure described by Zhao et al. [25,26,27], was supposed to be composed of a monolayer or fewer layers. There are two characteristic peaks at 2θ = 34° and 2θ = 60°, which correspond to (012) and (110) crystal planes of NiTi-LDH, respectively [29,30]. The characteristic peak of CNTs at 26° is retained for the NiTi-LDH@CNT material, indicating successful synthesis of the composite material.

Fourier transform infrared spectroscopy of NiTi-LDH, NiTi-LDH@CNTs and CNTs are shown in Figure 1b. The characteristic peaks at around 3435 cm^−1^ and 1631 cm^−1^ correspond to the stretching vibration of O−H bonds caused by surface adsorption of water and interlaminar crystalline water. The peak at 1386 cm^−1^ is attributed to the symmetric stretching vibration of $CO_{3}^{2-}$ ions in the NiTi-LDH interlayer [29,30]. The broad band at around 700 cm^−1^ corresponds to the M–O (metal–oxygen) stretching and bending vibrations, proving the formation of NiTi-LDH@CNT materials [19,20,21,22,23,24]. The M–O stretching and bending vibrations can be more easily discerned in the Raman spectrum of NiTi-LDH@CNT with an obvious absorption band at 689 cm^−1^ (Figure S1). Meanwhile, the weak peaks observed at 2923 cm^−1^ and 2850 cm^−1^ are associated with the stretching vibrations of CH_2_ and CH_3_. These two peaks should be related to the adsorption of some dodecyl sulfate anion (sodium dodecyl sulfate was used as surfactant in this work) on the surface of NiTi-LDH [26,27].

**Figure 2:**
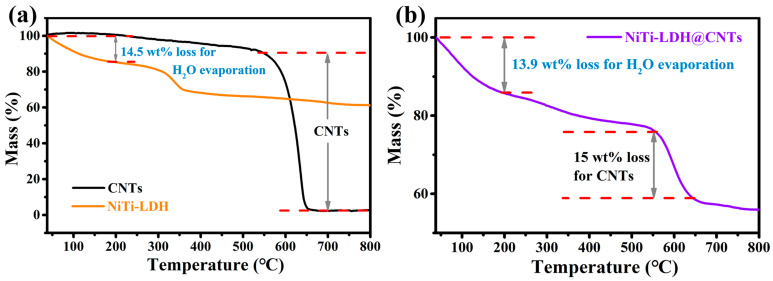
Thermogravimetric analysis (TGA) for (**a**) CNT, NiTi-LDH, and (**b**) NiTi-LDH@CNT composite samples.

Thermogravimetric analyses were conducted on NiTi-LDH, CNTs, and NiTi-LDH@CNTs, and the results are presented in Figure 2. The water contents for the NiTi-LDH and NiTi-LDH@CNTs are determined by the mass loss at 200 °C to be 14.5 wt% and 13.9 wt%, respectively [20]. The weight loss at around 350 °C for the NiTi-LDH is attributed to the removal of the hydroxyl group on the NiTi-LDH layer plate, as well as the interlayer cyanate and carbonate anions [31]. The content of CNTs in the NiTi-LDH@CNT composite was calculated by the mass loss in the temperature range of 550–650 °C to be approximately 15 wt%. The carbon content is consistent with the result obtained from the elemental analysis (Table S1).

**Figure 3:**
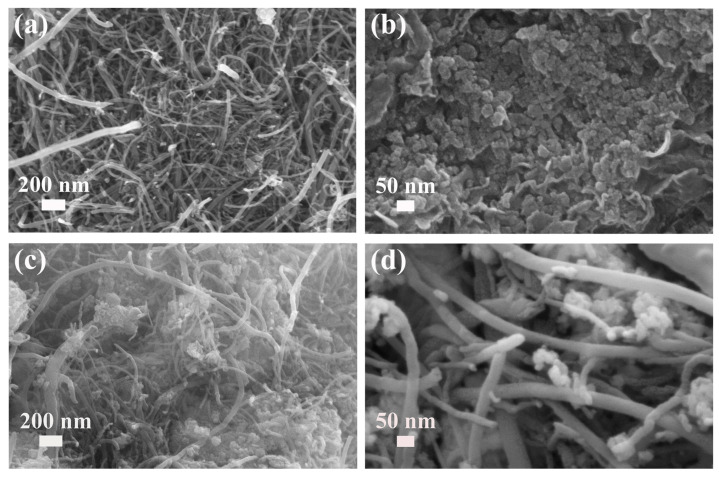
FESEM images of (**a**) CNTs, (**b**) NiTi-LDH and (**c**,**d**) NiTi-LDH@CNT composite samples.

Field-emission scanning electron microscopy (FE-SEM) was used to observe the morphology of the prepared NiTi-LDH, CNT, and NiTi-LDH@CNT composites. The CNTs with a diameter less than 50 nm and a length of several micrometers are entangled with each other (Figure 3a). The FE-SEM image of NiTi-LDH (Figure 3b) shows that many nanoparticles assembled with each other to form an agglomerate morphology. NiTi-LDH nanoflake with thickness of around 10 nm was also discerned. By comparing the FE-SEM image of blank CNTs and the FE-SEM images of NiTi-LDH@CNT composite (Figure 3c,d), it can be seen that NiTi-LDH nanoparticles are attached to CNTs and compound well with CNTs. 

**Figure 4:**
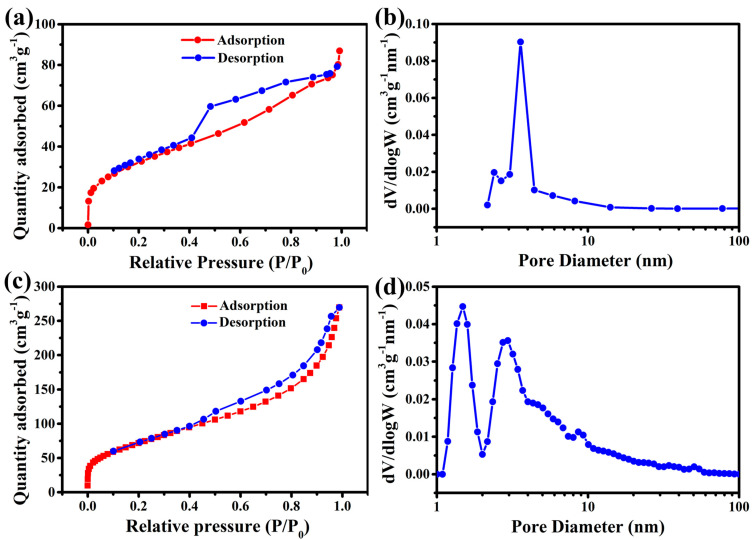
Nitrogen adsorption/desorption isotherms and the corresponding pore size distributions of as-prepared of (**a**,**b**) NiTi-LDH and (**c**,**d**) NiTi-LDH@CNT materials.

The nitrogen adsorption/desorption isotherms and pore size distributions of the prepared NiTi-LDH and NiTi-LDH@CNTs are shown in Figure 4. Both samples display typical type-IV isotherms (Figure 4a,c), demonstrating a mesoporous structure. The detailed parameters are listed in Table 2. The specific surface area and pore volume of the NiTi-LDH sample are 118 m^2^ g^−1^ and 0.12 cm^3^ g^−1^, respectively. When NiTi-LDH was compounded with CNTs, the specific surface area and pore volume of the composite increased to 266 m^2^ g^−1^ and 0.42 cm^3^ g^−1^, respectively. Based on the pore size distribution curves (Figure 4b,d), the average pore size of the NiTi-LDH@CNT composite and NiTi-LDH are 6.3 nm and 4.6 nm, respectively.

### 3.2. Electrochemical Performances

**Figure 5:**
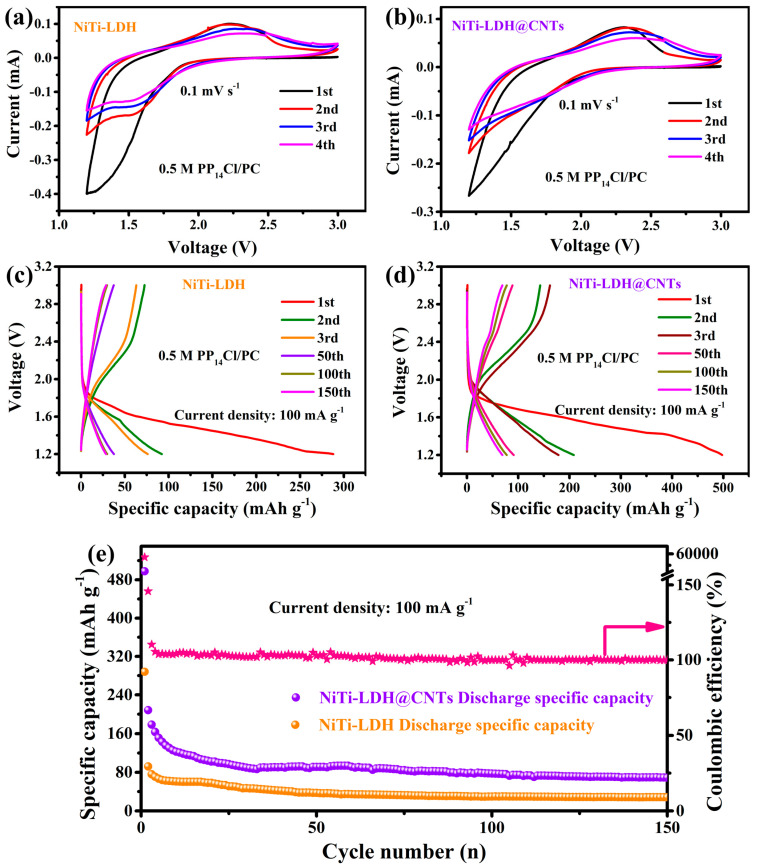
Cl¯ storage properties of NiTi-LDH and NiTi-LDH@CNT cathodes. (**a**,**b**) CV curves for the first four cycles at a scan rate of 0.1 mV s^−1^; (**c**,**d**) Discharge and charge curves at 100 mA g^−1^; (**e**) Coulombic efficiency and cycling performance at 100 mA g^−1^.

The Cl¯ ion storage performance of NiTi-LDH and NiTi-LDH@CNTs were investigated with LiCl/Li as anode and 0.5 mol L^−1^ PP_14_Cl/PC as electrolyte. LiCl/Li anode was prepared by fully discharging the assembled Li‖FeOCl coin cell. During this process, Cl ions migrate from FeOCl throughout the electrolyte to the metallic Li and react with the metallic Li to form LiCl. Since metallic Li is in excess, the LiCl/Li anode was therefore obtained. Figure S2 displays XRD and EDS maps of the as-prepared FeOCl, which are in agreement with our previous reports [16,32]. Moreover, Figure S3 displays the flower-like morphology of FeOCl. The successful preparation of LiCl/Li anode was confirmed by the XRD pattern as displayed in Figure S4b. Figure 5a,b depicts the CV curves of NiTi-LDH and NiTi-LDH@CNT electrodes in the voltage range of 1.2–3 V at 100 mA g^−1^. The CV curves of both electrodes exhibit an almost rectangular shape with no obvious redox peaks, implying a dominant capacitive storage mechanism [33]. The oxidation peak at 2.3 V corresponds to Cl^−^ entering the LDH interlayer, and the reduction peak at 1.4 V corresponds to Cl^−^ coming out of the LDH interlayer. Consistent with the CV curves, the galvanostatic charge and discharge curves for both electrodes (Figure 5c,d) displayed no obvious charge–discharge plateaus. The initial discharge specific capacity for NiTi-LDH and NiTi-LDH@CNT composites are 288 mAh g^−1^ and 498 mAh g^−1^, respectively. The addition of CNTs in the composites resulted in a higher reversible specific capacity of 69 mAh g^−1^ after 150 cycles, compared to 28 mAh g^−1^ of pure NiTi-LDH (Figure 5e). The NiTi-LDH@CNT electrode has a larger specific surface area and higher electronic conductivity, which makes it render a higher discharge capacity. In addition, the rapid capacity decay during the first 20 cycles for the NiTi-LDH@CNT electrode may be ascribed to its large surface area, which facilitates the formation of more SEI layer due to the increased side reaction with the electrolyte during continuous cycling. Furthermore, Coulombic efficiency of NiTi-LDH@CNT electrodes can reach 99%. It is worth noting that the capacity contribution from CNTs can be negligible (Figure S5).

**Figure 6:**
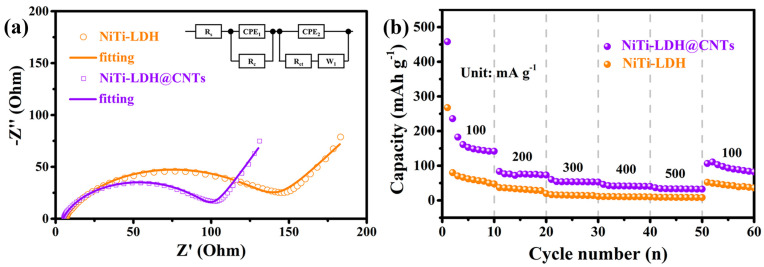
(**a**) Nyquist plots of the as-prepared NiTi-LDH and NiTi-LDH@CNT cathodes; (**b**) Comparison of rate capability between NiTi-LDH and NiTi-LDH@CNTs.

In order to study the electrochemical kinetics of pure NiTi-LDH and NiTi-LDH@CNT electrodes, EIS measurements were performed. The resulting Nyquist curves (Figure 6a) display arcs and straight lines at high and low frequencies, respectively. The arc at high frequency represents the combined contribution from the contact resistance and the charge transfer resistance. The equivalent circuit model shown in the inset of Figure 6a was used to fit the EIS plots. The fitting results are listed in Table 3. *R*_c_ and *R*_ct_ are contact resistance and charge transfer resistance, respectively. CPE1 and CPE2 are constant phase elements, *W*_1_ is Warburg impedance, and *R*_S_ is solution resistance. The *R*_ct_ value of the pure NiTi-LDH electrode was 136 Ω, while the *R*_ct_ value of NiTi-LDH@CNTs was 95 Ω. The smaller *R*_ct_ value of NiTi-LDH@CNTs indicates a faster charge transfer. Moreover, the higher the slope of the straight line at low frequency is, the smaller the diffusion resistance is. Compared with NiTi-LDH, a higher slope of the straight line for the NiTi-LDH@CNT electrode signifies an enhanced ion diffusion rate.

The rate performance of pure NiTi-LDH and NiTi-LDH@CNT electrodes were further studied. As shown in Figure 6b, the reversible specific capacities of NiTi-LDH@CNT composites at 100, 200, 300, 400 and 500 mA g^−1^ are 191, 74, 54, 42 and 33 mAh g^−1^, respectively, which are higher than 81, 31, 15, 11 and 9 mAh g^−1^ of pure NiTi-LDH. When the current density returns to 100 mA g^−1^ again, the average discharge specific capacity of the NiTi-LDH@CNT cathode is 94 mAh g^−1^, while the average discharge specific capacity of pure NiTi-LDH is 43 mAh g^−1^. The enhancement of rate performance for the NiTi-LDH@CNT cathode could be attributed to the improved conductivity caused by the introduction of carbon nanotubes.

### 3.3. Analysis of Chlorine Storage Mechanism

**Figure 7:**
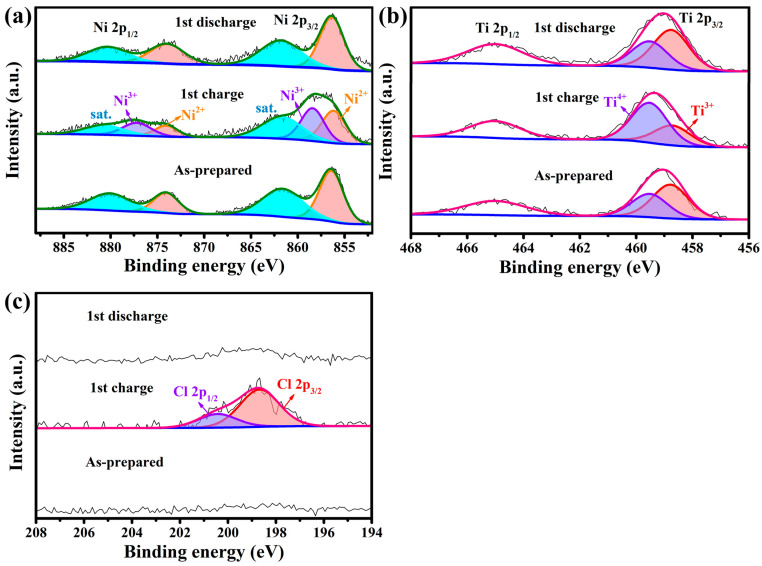
XPS spectra of Ni 2p (**a**), Ti 2p (**b**), and Cl 2p (**c**) for NiTi-LDH@CNT electrodes in various electrochemical states.

X-ray photoelectron spectra (XPS) were performed to investigate the chlorine storage mechanism during the charging and discharging process of NiTi-LDH@CNT composite electrodes. In Figure 7a, a distinct pair of Ni 2p_1/2_ and Ni 2p_3/2_ peaks in the Ni 2p XPS spectrum of the as-prepared electrode appeared, along with the satellite peaks at 880.4 eV and 861.8 eV. [20,22]. Upon charging, a new Ni 2p peak doublet located at higher binding energies of 877.1 and 858.4 eV emerged, which can be assigned to Ni^3+^ species [27,34,35,36]. Upon the following discharge, the Ni 2p signals belonging to the Ni^3+^ species disappeared. In Figure 7b, two pairs of distinct Ti 2p_1/2_ and Ti 2p_3/2_ peaks are visible in the Ti 2p XPS spectrum. Deconvolution peaks of Ti 2p_3/2_ at 459.5 eV and 458.7 eV are assigned to Ti^4+^ and Ti^3+^ states, respectively [24]. During the charging process, the signals of Ti^4+^ intensified while the signals of Ti^3+^ weakened, indicating that the average valence of Ti increased [37,38]. During the discharging process, the relative intensity of Ti^3+^ and Ti^4+^ signals were restored to the original state. In Figure 7c, the Cl 2p peak doublet at 198.7 (Cl 2p_3/2_) and 200.5 eV (Cl 2p_1/2_) came out during the charge process, providing proof for the Cl¯ entering the LDH layer [16,39]. The reversal of this change demonstrates the excellent oxidation/reduction reversibility of NiTi-LDH@CNTs as a CIB cathode material.

**Figure 8:**
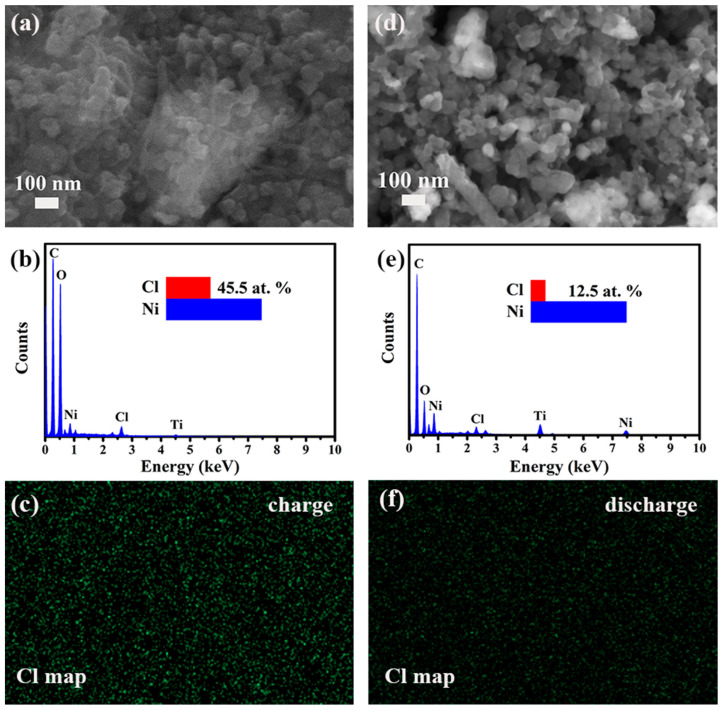
FE-SEM images and the corresponding EDS results of the NiTi-LDH@CNT cathodes: (**a**–**c**) 10th charge and (**d**–**f**) 10th discharge. The inset in (**b**,**e**) is the atomic ratio of Cl/Ni.

Figure 8 depicts the FE-SEM images and the corresponding energy-dispersive X-ray spectroscopy (EDS) results of the NiTi-LDH@CNT cathode during the 10th cycle. The EDS data (Figure 8b,e) reveals that there is an increase in the Cl element content during charging, indicating that chloride ions are inserted into the LDH gallery. During discharge, the Cl element content decreases, suggesting that the Cl¯ ions are extracted from the LDH gallery. The higher brightness of Cl mapping for the charged sample over the discharged sample further verified the insertion/extraction of Cl into/from the LDH interlayers.

**Figure 9:**
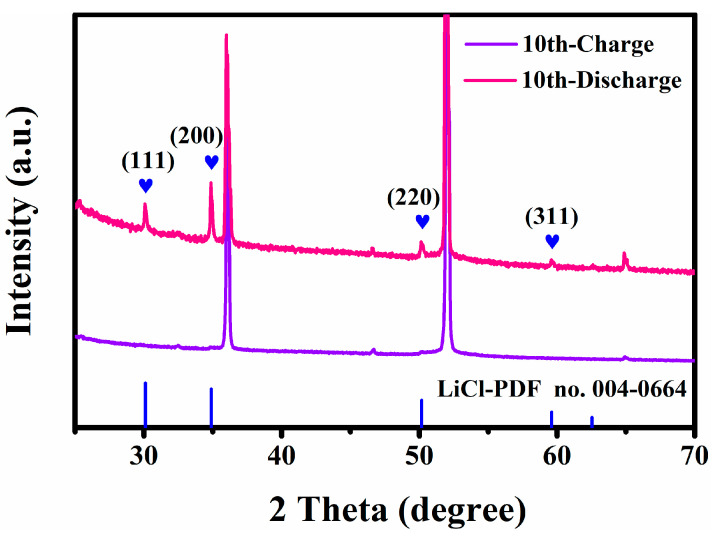
XRD patterns of anode side at 100 mA g^−1^ in the 10th cycle.

In addition, we have conducted ex-situ XRD studies on the anode side during the 10th cycle and the results are presented in Figure 9. Upon fully charging to 3 V, the characteristic diffraction peak of LiCl disappeared, indicating the release of Cl¯ from Li metal [20]. Upon fully discharging to 1.2 V, the characteristic diffraction peak of LiCl appeared, signifying the migration of Cl¯ back to the anode side to form LiCl. These findings confirm the reversible shuttle of chloride ions between cathode and anode.

## 4. Conclusions

CIBs have been regarded as one of the alternatives to conventional lithium-ion batteries due to the natural abundance of chloride resources in seawater, dendrite-free anodes upon cycling and high theoretical volumetric energy density. In this work, NiTi-LDH@CNT composite has been prepared by a reverse microemulsion method. We preliminarily investigated its chloride ion storage performance. When coupled with LiCl/Li anode, the NiTi-LDH@CNT composite cathode in PP_14_Cl/PC electrolyte delivered a reversible specific capacity of 69 mAh g^−1^ after 150 cycles at a current density of 100 mA g^−1^. The incorporation of CNTs significantly improved the electronic conductivity and dispersity of electroactive material, resulting in better electrochemical performance. The electrochemical mechanism was comprehensively revealed by ex-situ XPS, EDS and ex-situ XRD. This work provides an innovative avenue for the design of CIB cathode materials. Finally, although CIBs are one of the promising energy storage devices, it should be mentioned that there is still a potential risk of release of toxic chlorine gas upon overcharge from the practical point of view.

## Figures and Tables

**Table 1:** Electrochemical performance of reported LDHs as a Cl^−^ storage electrode in CIB.

Cathode Materials	Electrolyte	Current Density	Cycling Performance	References
**CoFe-Cl LDH**	0.5 M BpyCl-PP_14_TFSI-PC	100 mA g^−1^	160 mAh g^−1^ after 100 cycles	[19]
**NiMn-Cl LDH**	0.5 M Bpy_14_Cl-PC	50 mA g^−1^	130 mAh g^−1^ after 150 cycles	[20]
**Ni_2_V_0.9_Al_0.1_-Cl LDH**	1 M Bpy_14_Cl-PP_14_TFSI-PC	200 mA g^−1^	113.8 mAh g^−1^ after 1000 cycles	[21]
**NiFe-Cl LDH**	0.5 M Bpy_14_Cl-PC	100 mA g^−1^	130 mAh g^−1^ after 100 cycles	[22]
**CoNi-Cl LDH**	0.5 M Bpy_14_Cl-PC/[PP_14_][NTf_2_]	200 mA g^−1^	83 mAh g^−1^ after 50 cycles	[23]
**NiTi-Cl LDH**	0.5 M PP_14_Cl in PC	200 mA g^−1^	131.8 mAh g^−1^ after 200 cycles	[24]
**This work**	0.5 M PP_14_Cl in PC	100 mA g^−1^	69 mAh g^−1^ after 150 cycles	

**Table 2:** Physical data for the as-prepared NiTi-LDH and NiTi-LDH@CNT materials.

Samples	Total Pore Volume (cm^3^ g^−1^)	Average Pore Diameter (nm)	BET Specific Surface Area (m^2^ g^−1^)
**NiTi-LDH**	0.12	4.6	118
**NiTi-LDH@CNTs**	0.42	6.3	266

**Table 3:** Fitted impedance parameters for the as-prepared NiTi-LDH and NiTi-LDH@CNT cathodes.

Sample	*R*_s_ (Ω)	*R*_c_ (Ω)	*R*_ct_ (Ω)	*CPE*_1_ *-T*	*CPE*_1_ *-P*	*CPE*_2_ *-T*	*CPE*_2_ *-P*	*W*_1_ *-R*	*W*_1_ *-T*	*W*_1_ *-P*
**NiTi-LDH**	6.55	22.82	136	2.03 × 10^−5^	0.86	2.50 × 10^−5^	0.81	749.2	4.02	0.65
**NiTi-LDH@CNTs**	4.91	7.37	95	1.71 × 10^−5^	0.92	1.61 × 10^−5^	0.85	142	0.42	0.64

## Data Availability

Not applicable.

## Supplementary Materials

The following supporting information can be downloaded at: https://www.mdpi.com/article/10.3390/nano13202779/s1, Figure S1: Raman spectra; Figure S2 and Figure S3: XRD, EDS spectrum and FESEM images of FeOCl. Figure S4: XRD pattern of the as-prepared LiCl anode; Figure S5: (a) The charge and discharge curves of the blank CNTs and (b) the corresponding cycling performance at 100 mA g^−1^; Table S1: elemental analysis.
