# Supplementary material for: NiTi-Layered Double Hydroxide@Carbon Nanotube as a Cathode Material for Chloride-Ion Batteries
Source: Nanomaterials (Basel). 2023 Oct 17;13(20):2779. doi: 10.3390/nano13202779 (PMC10609344; doi:10.3390/nano13202779)
Supplement: Supplementary file 1 [file nanomaterials-13-02779-s001.zip › nanomaterials-2588713-supplementary.pdf]

# Supporting Information

# NiTi-Layered Double Hydroxide@Carbon Nanotube as a Cathode Material for Chloride-Ion Batteries

**Lu Zou [1], Shijiao Sun [1,*], Chang Zhang [1] and Xiangyu Zhao [1,2]**

[1] State Key Laboratory of Materials-Oriented Chemical Engineering, College of Materials Science and Engineering, Nanjing Tech University, Nanjing 211816, China; 202061203195@njtech.edu.cn (L.Z.); 202162103016@njtech.edu.cn (C.Z.); xiangyu.zhao@njtech.edu.cn (X.Z.)

[2] Jiangsu Collaborative Innovation Center for Advanced Inorganic Functional Composites, Nanjing Tech University, 30 Puzhu South Road, Nanjing 211816, China

* Correspondence: sunshijiao@njtech.edu.cn

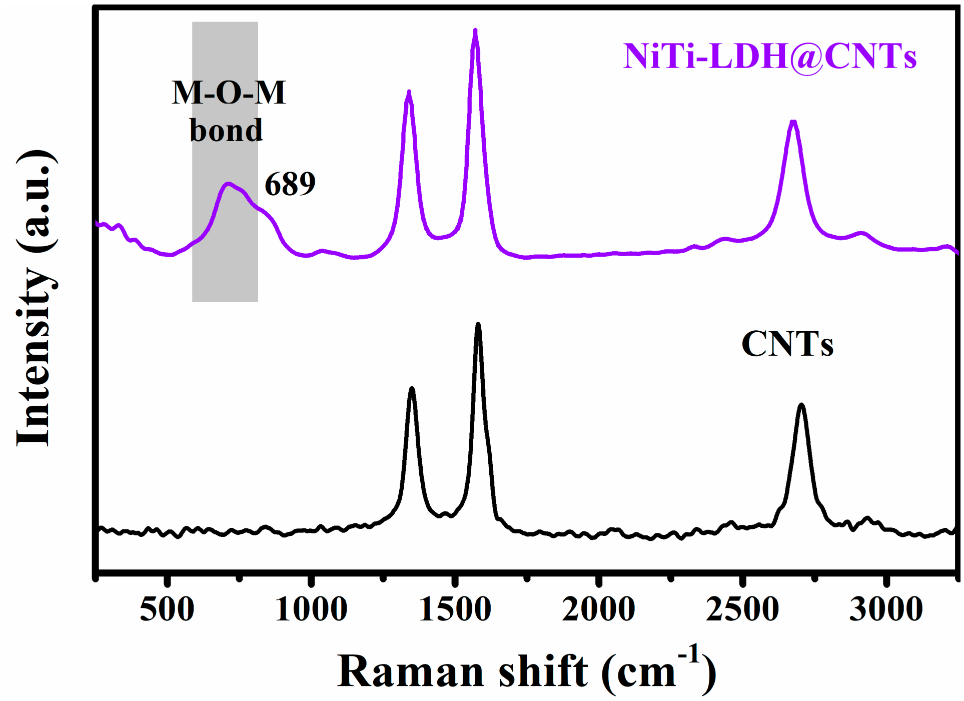


**Figure S1.** Raman spectra of blank CNTs and the as-prepared NiTi-LDH@CNT.

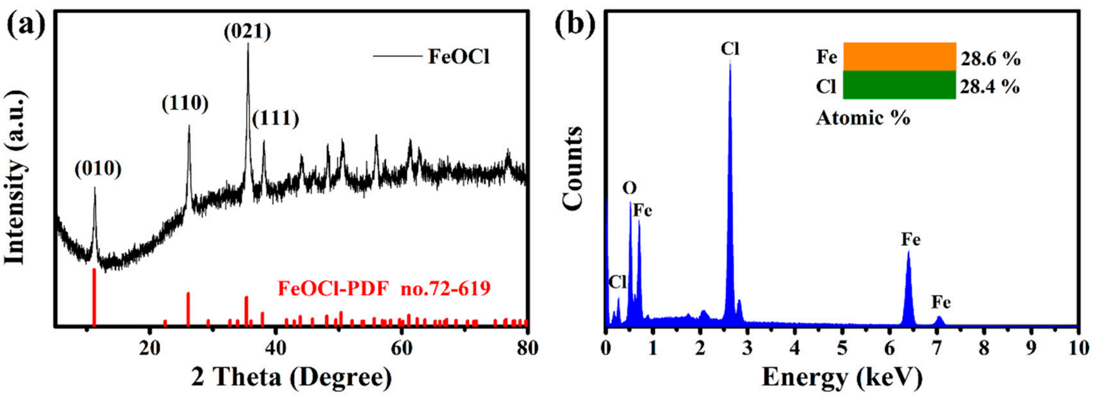


**Figure S2.** (a)XRD patterns and (b)EDS spectra of the of FeOCl

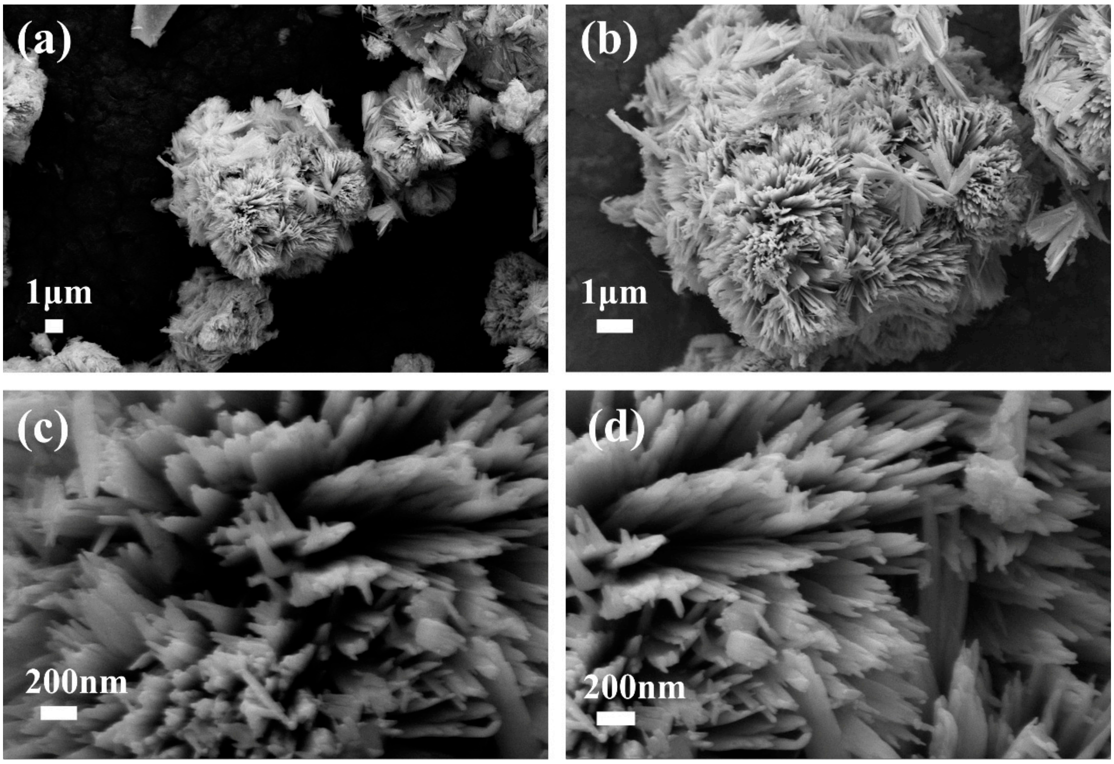


**Figure S3.** Field-emission scanning electron microscopy (FESEM) images of FeOCl prepared by a thermal decomposition method

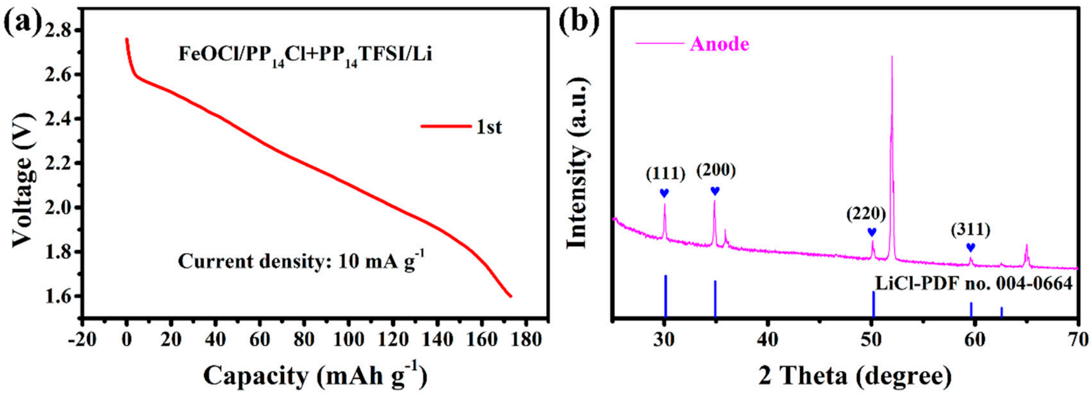


**Figure S4.** (a) Galvanostatic discharge curve of the Li‖FeOCl battery; (b) XRD patterns of the as-prepared LiCl anode

**Table S1.** CHNS elemental analysis for NiTi-LDH and NiTi-LDH@CNTs

| Sample | Element content (wt. %) | | |
|---|---|---|---|
| | C | H | S |
| **NiTi-LDH** | 6.32 | 3.70 | 1.09 |
| **NiTi-LDH@CNTs** | 21.25 | 2.97 | 1.99 |

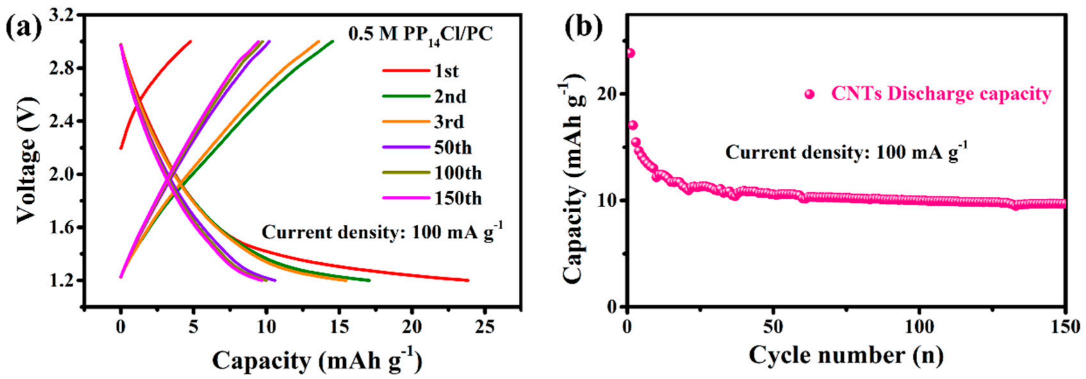


**Figure S5.** (a)The charge and discharge curves of the blank CNTs and (b) the corresponding cycling performance at 100 mA $g^{-1}$
